# Genome-resolved insights into a novel *Spiroplasma* symbiont of the Wheat Stem Sawfly (*Cephus cinctus)*

**DOI:** 10.7717/peerj.7548

**Published:** 2019-08-27

**Authors:** Carl J. Yeoman, Laura M. Brutscher, Özcan C. Esen, Furkan Ibaoglu, Curtis Fowler, A. Murat Eren, Kevin Wanner, David K. Weaver

**Affiliations:** 1 Department of Animal & Range Sciences, Montana State University, Bozeman, MT, United States of America; 2 Department of Microbiology & Immunology, Montana State University, Bozeman, MT, United States of America; 3 Department of Medicine, University of Chicago, Chicago, IL, United States of America; 4 Marine Biological Laboratory, The Josephine Bay Paul Center for Comparative Molecular Biology and Evolution, Woods Hole, Massachuetts, United States of America; 5 Department of Plant Sciences & Plant Pathology, Montana State University, Bozeman, MT, United States of America; 6 Department of Land Resources and Environmental Sciences, Montana State University, Bozeman, MT, United States of America

**Keywords:** *Spiroplasma*, Scanning electron microscopy, Wheat stem sawfly, Metagenomics, Microbial genomics, Pangenomics, Phylogenomics

## Abstract

Arthropods often have obligate relationships with symbiotic microbes, and recent investigations have demonstrated that such host-microbe relationships could be exploited to suppress natural populations of vector carrying mosquitos. Strategies that target the interplay between agricultural pests and their symbionts could decrease the burden caused by agricultural pests; however, the lack of comprehensive genomic insights into naturally occurring microbial symbionts presents a significant bottleneck. Here we employed amplicon surveys, genome-resolved metagenomics, and scanning electron microscopy to investigate symbionts of the wheat stem sawfly (*Cephus cinctus*), a major pest that causes an estimated $350 million dollars or more in wheat yield losses in the northwestern United States annually. Through 16S rRNA gene sequencing of two major haplotypes and life stages of wheat stem sawfly, we show a novel *Spiroplasma* species is ever-present and predominant, with phylogenomic analyses placing it as a member of the ixodetis clade of mollicutes. Using state-of-the-art metagenomic assembly and binning strategies we were able to reconstruct a 714 Kb, 72.7%-complete *Spiroplasma* genome, which represents just the second draft genome from the ixodetis clade of mollicutes. Functional annotation of the *Spiroplasma* genome indicated carbohydrate-metabolism involved PTS-mediated import of glucose and fructose followed by glycolysis to lactate, acetate, and propionoate. The bacterium also encoded biosynthetic pathways for essential vitamins B2, B3, and B9. We identified putative *Spiroplasma* virulence genes: cardiolipin and chitinase. These results identify a previously undescribed symbiosis between wheat stem sawfly and a novel *Spiroplasma* sp., availing insight into their molecular relationship, and may yield new opportunities for microbially-mediated pest control strategies.

## Introduction

The wheat stem sawfly (*Cephus cinctus*) is a hymenopteran insect native to the Western U.S. and Canada. Currently, wheat stem sawflies (further referred to as WSS) are some of the worst pests of wheat production (Shanower & Hoelmer, 2004). WSS causes losses in excess of $350 million US annually (Beres et al., 2011). Females oviposit into wheat stems and, after hatching the larvae consume the internal structures lining the stem, both diminishing photosynthetic capacity of the plant and making it more susceptible to lodging (Shanower & Hoelmer, 2004; Beres et al., 2011). Current methods used by growers to reduce crop losses due to WSS include operational control measures (e.g., crop rotation, tillage, and swathing), infrequent use of insecticides, and biological control agents (e.g., parasitic wasps) (Shanower & Hoelmer, 2004; Beres et al., 2011). Most research efforts aimed at curbing this problem are to develop WSS-resistant wheat cultivars, including solid-stemmed varieties that provide less suitable habitat for WSS reproduction, but many of these wheat varieties have lower yield potentials and inconsistent pith expression, so the identification of alternative controls and other host plant resistance is desirable (Beres, Cárcamo & Byers, 2007; Beres et al., 2011; Knodel et al., 2009; Portman et al., 2018).

One option may be to manipulate symbioses between WSS and any symbiotic microbe(s) to negatively affect the insects’ fitness. Metabolic contributions of symbiotic microbes are often critical to the nutritive, physiological, immunological, reproductive, and developmental health of animals (Torrazza & Neu, 2011; Flint et al., 2012; Thaiss et al., 2016; Borgogna & Yeoman, 2017). Insects, in particular, have many well-described long-term relationships with their microbiota that include obligate-(endo)symbioses necessary for host (and microbe) survival and reproductive success (Moran & Telang, 1998; Wernegreen, 2002). *Wolbachia* and *Spiroplasma* are the most prevalent and characterized genera of insect-associated symbionts. *Wolbachia* are Gram-negative, intracellular, members of the Rickettsial family that are maternally transmitted in at least 40% of arthropod species (Zug & Hammerstein, 2012). Many *Wolbachia*-insect interactions are parasitic, reducing host lifespan and affecting reproductive phenotypes, causing outcomes such as feminization and male killing of larvae, parthenogenesis, and cytoplasmic incompatibility (Werren, Baldo & Clark, 2008). Because *Wolbachia* have also been reported to reduce viral replication in their hosts they have been explored as a potential biocontrol for virus-vectoring mosquito populations (Teixeira, Ferreira & Ashburner, 2008; Mousson et al., 2012). A specimen of WSS from Alberta assessed for presence of *Wolbachia* tested negative (Floate, Kyei-Poku & Coghlin, 2007). *Spiroplasma,* conversely, are small, helical, motile bacteria lacking cell walls (Anbutsu & Fukatsu, 2011). Most *Spiroplasma* species are considered commensal, but others have been shown to induce male-killing in their insect host or cause diseases in plants (e.g., Corn Stunt) after transfer by the vector (Chen & Liao, 1975; Regassa & Gasparich, 2006; Haselkorn, 2014; Bolaños, Servín-Garcidueñas & Martínez-Romero, 2015). Some *Spiroplasma* spp. have also been reported to be mutualistic, improving host defenses against fungal pathogens (Łukasik et al., 2012), parasitic nematodes (Jaenike et al., 2010), and parasitoid wasps (Xie, Vilchez & Mateos, 2010; Xie et al., 2013). *Spiroplasma* species are grouped into three phylogenetic clades: Citri-Chrysopicola-Mirum (CCM), Apis, and Ixodetis (Gasparich et al., 2004).

Herein, we sought to identify the symbiotic microbes of WSS and obtain insight into the nature of the insect-microbe relationships so that we could begin to determine if these insect-microbial relationships could be exploited as alternate measures to control WSS damage in crops.

## Materials and Methods

### Insect collection

Midseason haplodiploid larval (*n* = 10) and early-adult WSS (*n* = 10) representatives were collected from highland grasses (Flesher pass, Lincoln, MT; *n* = 10) and lowland wheat crops (Three Forks, MT; *n* = 10) over the 2013, 2015, and 2016 growing seasons. Together, the highland grass (Mountain) and lowland wheat (Northern) populations represent two of the three major haplotype clades of WSS (Lesieur et al., 2016). Northern sample collection was carried out on private lands with permission from the land owners and Mountain samples on public lands in compliance with existing regulations for insects defined as non-commercial, as determined by local regulatory offices.

### Sample preparation and DNA extraction

Specimens were surfaced sterilized by washing samples in 1% bleach and rinsing with an excess of sterile water under aseptic conditions. Twenty adult females and whole larvae were individually processed through a Mo-Bio Power-Soil kit (Mo-Bio, Carlsbad, CA, USA) following the manufacturers protocol with inclusion of a 1 min bead-beating step performed in a Mini-Beadbeater-96 (Biospec products; Bartlesville, OK, USA) at 2,400 oscillations/min and using the 0.7-mm garnet beads supplied with the Mo-Bio kit instead of the 10 min vortex. An additional collection of 50 lowland WSS larvae collected in 2016 were processed exclusively for metagenomic sequencing to increase the representation of the *Spiroplasma* sp. in the DNA preparation and final sequencing data. This was deemed necessary because 98.9 ± 1.0% of each sample’s reads from whole processed individuals were classified as being eukaryotic, and included host reads (*Cephus cinctus*) and reads belonging to wheat (*Triticum aestivum*). The protocol proceeded as follows: Following washing, as above, the 50 lowland WSS larvae were lightly crushed with a mortar and pestle in 3 ml sterile PBS under aseptic conditions. The entire solution was filtered through 450 nm nitrocellulose filters to enrich the bacteria in the cell lysate for *Spiroplasma* and remove other bacterial species larger than 450 nm, as per Nai et al. (2014). The filtrate was then centrifuged at 16,000× g for 30 min at 4 °C in order to pellet the cells that passed through the filter. The pellet was resuspended, filtered, and centrifuged again. The final pellet was processed through a Mo-Bio Power-Soil kit (Mo-Bio), as described above. This extraction procedure yielded 15 ng total DNA that, following sequencing and retrospective analyses, was found to have reduced the total eukaryotic DNA to 89.9%. This sequence data was used for metagenomic assembly but did not provide *Spiroplasma* sp. genomic data that was not otherwise captured in metagenomes of individual insects or larvae.

### 16S rRNA gene sequencing

The V3 and V4 hypervariable regions of the 16S rRNA gene was PCR amplified with previously described dual indexed primers (Yeoman et al., 2018). The PCR was performed using KAPA HiFi DNA Polymerase (Kapa Biosystems, Wilmington, MA, USA) and a PCR protocol with initial denaturation of 98 °C for 45 s, followed by 25 cycles of 15 s denaturation at 98 °C, 30 s annealing step at 60 °C, and 30 s elongation step at 72 °C, and then a final extension at 72 °C for 2 min. The PCR reaction was limited to 25 cycles to minimize the potential for chimera formation and error (Smyth et al., 2010). PCR amplicon concentrations and quality were checked on a Bioanalyzer TapeStation using D1000 Screentapes (both Agilent Technologies, Santa Clara, CA, USA). Resulting amplicons were pooled at equimolar concentrations, cleaned up using the AxyPrep MagTM PCR Clean-up Kit (Corning, NY, USA), and then gel purified from a 1.5% agarose gel using the QIAquick gel extraction kit (QIAGEN, Valencia, CA, USA) according to kit instructions. The 16S rRNA gene library was then quantified using the KAPA Library Quantification Kit (Kapa Biosystems), diluted to 15 pM, mixed with 10% phiX and sequenced on an Illumina MiSeq using a 2 × 250 V2 sequencing kit (Illumina, San Diego, CA, USA). On average, each sample yielded 4,125 reads with one sample only yielding 151 reads. Because the low yielding sample provided the same result as other samples, no further sequencing was attempted, and those results were retained.

### Microbial community structure analyses

Raw sequencing data were obtained in FASTQ format and assembled into contigs using the default settings of PANDASEQ (Masella et al., 2012) but with FASTQ output (‘-F’) to retain quality scores. Assembled contigs were then trimmed at the 3′ end from the first nucleotide with a *Q*-score of <20 using the fastq_quality_trimmer tool from FastX-Toolkit (http://hannonlab.cshl.edu/fastx_toolkit/links.html). Retained contigs with a sequence length of <200 nt were subsequently removed. Sequences were then processed using mothur (Schloss et al., 2009) and aligned to the Silva v119 reference database (Pruesse et al., 2007). Sequences were removed if they had homopolymers >10 nt, an ambiguous nucleotide, did not align over the V3–V4 region of the 16S rRNA gene, or were determined using chimera.uchime to be a chimera. To reduce the effect of sequencing error, the sequences were then pre-clustered based on 2% nearest neighbor approach, previously described (Huse et al., 2010). Sequences were then clustered into phylotypes based on taxonomic classification using the “cluster.split” command (splitmethod = classify), and classified by RDP Naive Bayesian Classifier (Cole et al., 2009). Microbial composition was compared among samples using multivariate statistical approaches provided in the vegan package of R (Oksanen et al., 2007).

### SEM sample preparation

Twenty-five larvae washed in 70% ethanol were lightly crushed with a mortar and pestle in 3 ml sterile PBS under aseptic conditions. The entire solution was filtered through a disposable 450 nm nitrocellulose filter (Nai et al., 2014), and pelleted by centrifugation at 16,000× g for 30 min at 4 °C as described above. The pellet was then eluted in 300 µl PBS. A volume of 40 µl of the pellet solution was pipetted onto a coverslip and left for one hour for bacteria to attach. For fixation, a modified protocol from Nai et al. (2014) was used where the cover slip was immersed in a 1.5% glutaraldehyde solution prepared in 0.1 M cacodylic acid buffer (pH 7.3) and incubated at 4 °C overnight. The coverslip was then rinsed in water for 20 min four times. The sample was then progressively dehydrated by placing the slip in solutions increasing by relative ethanol: 25% EtOH 30 min, 50% EtOH 30 min, 75% EtOH 30 min, 95% EtOH 30 min, 100% 60 min (three times to rinse coverslip off). Critical point drying, specimen mounting, and SEM imaging were performed at the Imaging and Chemical Analysis Laboratory (ICAL) at Montana State University. The coverslip was critically point dried in a Tousimis SAMDRI®-795 (Tousimis, Rockville, MD, USA) for thirty minutes, and mounted. The coverslip was then imaged on a Zeiss SUPRA 55VP (Carl Zeiss, Oberkochen, Germany) field emission scanning electron microscope.

### Metagenomic sequencing

DNA was prepared using a Nextera XT kit as per manufacturer’s instructions and resulted in ∼250 bp insert sizes. Metagenomic libraries were pooled in equimolar concentrations with three libraries per pool and sequenced on Illumina MiSeq sequencing platform using a 2 × 150 V2 sequencing kit.

After a preliminary round of analysis, it was determined that WSS genome sequence data deposited in NCBI (Robertson et al., 2018), also included sequence data from the *Spiroplasma* sp. Therefore, in an effort to improve our *Spiroplasma* genome data and assembly, the raw sequence files from the WSS genome sequencing effort were also downloaded and used.

### Metagenomic sequencing analysis

Raw sequencing data obtained in FASTQ format were filtered to remove low-quality reads using ‘iu-filter-quality-minoche’, a program in illumina-utils v1.4.1 (Eren et al., 2013). High-quality reads were assembled into longer contiguous segments of DNA (contigs) using MEGAHIT v1.0.6 (Li et al., 2015), and only contigs longer than 2,500 nt were kept for downstream analyses. Metagenome-assembled genomes (MAGs) were generated and curated using anvi’o v3 following a workflow outlined previously by Eren et al. (2015). Briefly, Bowtie2 (Langmead & Salzberg, 2012) mapped short metagenomic reads back to contigs using default parameters, and samtools (Li et al., 2009) converted resulting SAM files into sorted and indexed BAM files. We then used the program ‘anvi-gen-contigs-database’ to create a contigs database for the assembly, during which Prodigal v2.6.3 (Hyatt et al., 2010) predicted open-reading frames and HMMer identified of bacterial single-copy core genes among contigs. Then we used ‘anvi-profile’ to process BAM files from each metagenome, and merged resulting profile databases using ‘anvi-merge’. We then manually identified metagenomic bins in the anvi’o interactive interface using the hierarchical clustering of contigs based on patterns of tetra-nucleotide frequency and differential coverage patterns across metagenomes. The program ‘anvi-summarize’ generated a static HTML output, which gave access to FASTA files as well as coverage and detection statistics of each genome bin (and individual genes within them) across metagenomes. Comparative analysis of all bacterial single-copy core genes and their coverage were used to estimate genome completeness and redundancy, as previously described (Eren et al., 2015).

Three bins were determined, that phylogenomic analyses of all single copy core genes linked to either the host WSS, wheat, or to a bacterium of the order *Entomoplasmatales,* to which *Spiroplasma* belongs. Additional, phylogenetic and phylogenomic analyses described below confirmed this bin as belonging to a novel *Sprioplasma* sp. Reads that binned with the *Spiroplasma* sp*.* were annotated using the standard PATRIC pipeline (Wattam et al., 2013). Reads that binned with the WSS host were translated to their amino acid sequences in all frames using MEGA7 (Kumar, Stecher & Tamura, 2016) and then annotated to KEGG orthologies (KO) using BlastKOALA (Kanehisa, Sato & Morishima, 2016). Both WSS host and *Spiroplasma* sp. KO’s were then mapped to metabolic maps using iPATH v3 (Letunic et al., 2008) and analyzed to determine unique and overlapping pathways (Fig. S2). An explorable version of this pathway is available at https://pathways.embl.de/selection/ua1khKYbv9AKjZ8PimR.

### Pangenomic and phylogenomic analyses

To compute the *Spiroplasma* pangenome, we used the anvi’o pangenomic workflow outlined by Delmont & Eren (2018) using the anvi’o version v5.5 (Eren et al., 2015). A reproducible workflow for this analysis is available at the URL http://merenlab.org/data/spiroplasma-pangenome. Briefly, we identified and downloaded all *Spiroplasma* genomes from NCBI using the program ncbi-genome-download (available from https://github.com/kblin/ncbi-genome-download) using flags “–assembly-level complete bacteria –genus Spiroplasma” (Genome accessions and information is listed in Table S1), and stored the information about these genomes that is also reported by ncbi-genome-download into a text file. We then used the anvi’o program anvi-script-process-genbank-metadata with the flag “–exclude-gene-calls-from-fasta-txt”, added three additional MAGs recently generated for members of the Ixodetis clade of mollicutes (EntAcro1 and EntAcro10 (Sapountzis et al., 2018) as well as our *Spiroplasma* sp. WSS MAG) into the resulting external genomes file. We then run the program anvi-gen-genomes-storage using the external genomes file, and finally the program anvi-pan-genome on the resulting genomes storage to compute the pangenome. To perform a phylogenomic analysis to infer evolutionary associations between genomes, we identified the *Spiroplasma* single-copy core genes revealed by the pangenome and recovered their aligned amino acid sequences using the program anvi-get-sequences-for-gene-clusters with parameters “–min-num-genomes-gene-cluster-occurs 31 –max-num-genes-from-each-genome 1 –concatenate-gene-clusters”. We then used trimAl v1.4.rev22 (Capella-Gutierrez, Silla-Martinez & Gabaldon, 2009) to remove positions that were gaps in more than 50% of the genes in the alignment using the parameter “-gt 0.50”, and IQ-TREE v1.5.5 (Nguyen et al., 2014) with the ‘WAG’ general matrix model (Whelan & Goldman, 2001) to infer the maximum likelihood tree. We used the program anvi-import-misc-data to import the resulting tree into the anvi’o pan database, and program anvi-display-pan to visualize the output.

### Data availability

16S rRNA sequences were submitted to Sequence Read Archive and are available under accession number SRP108219. Metagenomic sequences were submitted to Sequence Read Archive under accession number SRP108220. Additional sequence data generated by Illumina HiSeq as part of the WSS genome project (Robertson et al., 2018) are available under accession number SRS694145. The metagenomic assembled genome is available in GenBank under bioproject PRJNA540284 and is available publicly at Patric with the genome ID 2132.146 (*Spiroplasma* sp. WSS).

## Results

### Wheat stem sawflies are colonized by a *Spiroplasma* sp

16S rRNA gene sequence profiles were obtained from individual whole-body WSS specimens representing two of the three major WSS haplotype clades, Northern and Mountain (Lesieur et al., 2016), and of both late stage larval and adult life stages. On average, each sample yielded 4,090 reads and across all samples these reads clustered into eight different phylotypes that each classified to unique bacterial genera. One phylotype that classified as a *Spiroplasma spp.,* was found to predominate all individuals and life stages and represented 88.5 ± 8.3% of all reads (Table 1). Other phylotypes were much less abundant and their observed presence varied among samples. Each of the less prevalent and abundant phylotypes were classified as genera from the family Pasteurellaceae, including *Nicoletella*, *Histophilus*, *Lonepinella*, *Actinobacillus*, *Basfia*, *Haemophilus*, and *Aggregatibacter* genera (Table 1).

### An α-helical bacterium consistent with *Spiroplasma* is observable in larval lysates via SEM imaging

**Figure 1:**
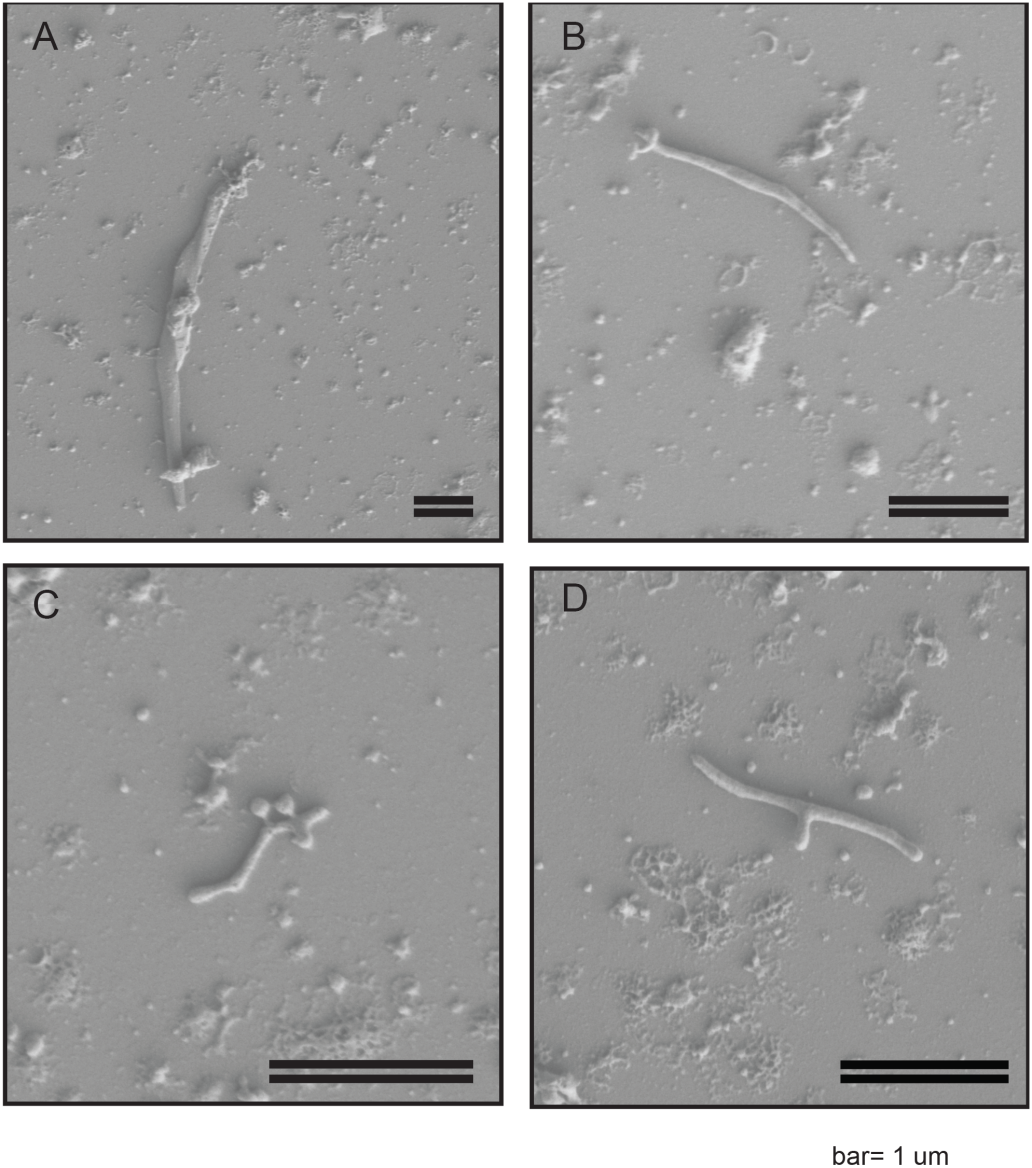
SEM images of WSS reveal non-helical *Spiroplasma*-like morphotype. Scanning Electron Microscopy Images of filtered WSS larvae lysates reveal a non-helical filamentous bacterium, consistent with *Spiroplasma*. Cells were non-helical and filamentous, ranging 1–4 µM in length (A–C). Some cells exhibited a *y*-shaped morphology, which may be cells replicating via longitudinal fission (D). The scale bar is 1 µM.

In order to confirm the presence of a *Spiroplasma* spp*.* in WSS, we employed a previously described protocol for the enrichment of *Spiroplasma* (Nai et al., 2014) to WSS larvae lysates and interrogated the resultant via Scanning Electron Microscopy (SEM). Cells were identified that had long filamentous shapes varying from 1–4 uM in length. These cells were not observed to be distinctly helical (Fig. 1) but were consistent with previously described *Spiroplasma* spp. morphologies. Some cells exhibited a y-shaped morphology (Fig. 1D), that has previously been observed in *Spiroplasma poulsonii* during division via longitudinal scission (Ramond et al., 2016).

### Metagenomic and genomic insight into the WSS *Spiroplasma* sp.

Attempts to cultivate the *Spiroplasma* sp. using previously described media for the cultivation of other *Spiroplasma* spp. were unsuccessful (for further discussion see Text S1). So, to gain insight into the genomic context of the WSS-associated *Spiroplasma* population, we used a genome-resolved metagenomics approach. On average sequencing of each individual WSS adult and larval specimen yielded ∼2.9 million reads per sample, which preliminary analyses determined to contain 98.9 ± 1.0% eukaryotic reads. A second separate preparation of 50 Northern WSS larvae following a previously described protocol to enrich for *Spiroplasma* spp. (Nai et al., 2014) yielded ∼20 million reads that were found to comprise 89.9% eukaryotic reads. Larvae were selected for this process because they were found to have greater DNA sequence representation of the *Spiroplasma* sp. in preliminary analyses of individual samples (0.21 ± 0.52% adults vs. 1.4 ± 0.83% larvae). The co-assembly of 12 metagenomes and the enriched preparation resulted in 14,217 contigs longer than 2,500 nt. The majority of these contigs matched to the host WSS genome, however, we were able to identify two additional genome bins among the rest of the contigs (Fig. S1). One of these bins matched to wheat DNA that was derived exclusively from cropland samples of WSS larvae. The second bin contained 145 contigs (max contig size of 15 kbp and an N_50_ of 5,160 bp) with a collective length of 713,566 bp. Analysis of all bacterial single-copy core genes also allowed us to estimate that this genome bin was 72.7% complete, and the sequence homology of the ribosomal proteins resolved it to *Spiroplasma*. The AT-rich *Spiroplasma* metagenome-assembled genome (MAG; subsequently referred to as *Spiroplasma* sp. WSS) had a GC% content of 24.56%, 754 open reading frames, components of a single ribosomal RNA operon, and 23 tRNA encoding genes (Full annotation at: https://www.patricbrc.org/view/Genome/2132.146 and NCBI GenBank accession VBWQ0100001 (Bioproject PRJNA540284)). Functional annotation of the 754 putative genes (427 of which were classified as hypothetical proteins) indicated that the bacterium is capable of importing and phosphorylating sugars via a phosphoenolpyruvate phosphotransferase (PTS) system (FCO83_02200 & FCO83_02960). Although specific substrates are not determinable, pathways for the utilization of glucose-6 phosphate (G6P), and fructose-6P are evident, including a complete glycolytic (FCO83_00750, FCO83_00765, FCO83_02365, FCO83_02370, FCO83_02375, FCO83_02525, FCO83_03295; Fig. S2) and a near-complete pentose phosphate pathway (FCO83_01715, FCO83_02845, & FCO83_03285; Fig. S2). Additionally, the bacterium encodes phosphomannomutase (FCO83_00995; EC 5.4.2.8) indicating mannose may also be a utilizable substrate, although no additional catabolic genes enabling the further processing of mannose-1P or mannose-6P were evident.

**Table 1:** Relative abundance of bacterial phylotypes from 16S rRNA survey. Numbers represent the average ± standard deviation of the relative abundance of each detected phylotype from five replicates of each life stage and haplotype. Where a phylotype was not detected in any replicate it is denoted as n.d.

**Genera**	**Mountain Larvae**	**Mountain Adult**	**Northern Larvae**	**Northern Adult**
*Spiroplasma*	85 ± 4.7%	92 ± 9.6%	92 ± 5.0%	85 ± 12%
*Actinobacillus*	0.3 ± 0.8%	0.9 ± 0.9%	0.4 ± 0.5%	*n.d*
*Aggregatibacter*	2.1 ± 2.3%	1.0 ± 0.7%	0.2 ± 0.4%	0.6 ± 1.3%
*Basfia*	0.2 ± 0.5%	*n.d.*	0.5 ± 0.7%	*n.d.*
*Haemophilus*	0.2 ± 0.5%	0.3 ± 0.6%	*n.d.*	*n.d.*
*Histophilus*	4.9 ± 2.7%	2.6 ± 4.7%	0.8 ± 1.8%	3.3 ± 4.1%
*Lonepinella*	0.9 ± 1.3%	0.7 ± 1.2%	0.3 ± 0.7%	0.6 ± 1.1%
*Nicoletella*	6.2 ± 2.6%	2.8 ± 3.2%	6.0 ± 3.8%	10 ± 7.7%

Genes for the production of lactate (FCO83_00760) and the short chain fatty acids acetate and propanoate suggest these are likely major fermentative end products. Additionally, a multi-functional formate efflux transporter (FCO83_02325 & FCO83_02330) may indicate formate is also a major end product. This transporter may also facilitate the export of each of these terminal acid products as has been seen in other bacterial species (Lü et al., 2012). The *Spiroplasma* sp. also encodes an F-type ATPase suggesting it is capable of oxidative phosphorylation and a low-affinity phosphate transport system (FCO83_00795, FCO83_00800, & FCO83_00805) for the scavenging of inorganic phosphate. The *Spiroplasma* sp. WSS MAG encodes near complete pathways for the biosynthesis of vitamins B2 (riboflavin), B3 (niacin & nicotinamide), and B9 (Folate). The presence of genes encoding thiamine-specific (ThiT; FCO83_03200) and folate-specific (FolT; FCO83_02260) ECF (energy coupling factor) importers (structural components include, FCO83_01665, FCO83_01670, FCO83_01675), and a ribosyl nicotinamide transporter (FCO83_00500) suggests the microbe can both biosynthesize and import folate and nicotinamide, as has been described in other bacterial species, but is likely dependent on extracellular levels of vitamin B1 (thiamine).

Seven ORFs that exhibited homology (39.7–53.9% amino acid identity) to the male-killing gene, Spaid of *S. poulsonii* (Harumoto & Lemaitre, 2018) were identified (Table 2). Each contained ankyrin repeat domains, as described for Spaid, but none possessed the deubiquitinase or N-terminal signal peptide domains also described by Harumoto & Lemaitre (2018). Finally, two additional potential virulence factors were identified, that included cardiolipin synthetase (FCO83_00175; EC 2.7.8.-) and chitinase (FCO83_02115; EC 3.2.2.27).

**Table 2:** Potential spaid homologues identified in the WSS-associated spiroplasma genome. Spiroplasma sp. WSS ORFs displaying homology to the S. poulsonii gene, Spaid, determined to be involved in the male-killing phenotype. Only Ankyrin repeat domains (IPR002110) were identified and the number of their domain repeats are indicated.

**Gene locus tag (Patric feature ID)**	***E*-value**	**% AA identity**	**Coverage of Spaid**	**Domains Identified**
FCO83_00980 (PEG.209)	2e^−16^	47.8%	100%	Ankyrin repeats^1^ (×5)
FCO83_02170 (PEG.469)	1e^−15^	50.8%	100%	Ankyrin repeats^1^ (×11)
FCO83_02755 (PEG.596)	1e^−14^	49.1%	100%	Ankyrin repeats^1^ (×8)
FCO83_00085 (PEG.21)	2e^−13^	53.9%	91%	Ankyrin repeats^1^ (×1)
FCO83_01420 (PEG.305)	5e^−13^	47.5%	100%	Ankyrin repeats^1^ (×7)
FCO83_00005 (PEG.1)	1e^−12^	52.6%	89%	Ankyrin repeats^1^ (×2)
FCO83_01210 (PEG.255)	3e^−07^	39.7%	91%	Ankyrin repeats^1^ (×3)

### Phylogenomic and phylogenetic analyses place the *Spiroplasma* sp. WSS in the *Ixodetis* Clade of *Spiroplasma* species

**Figure 2:**
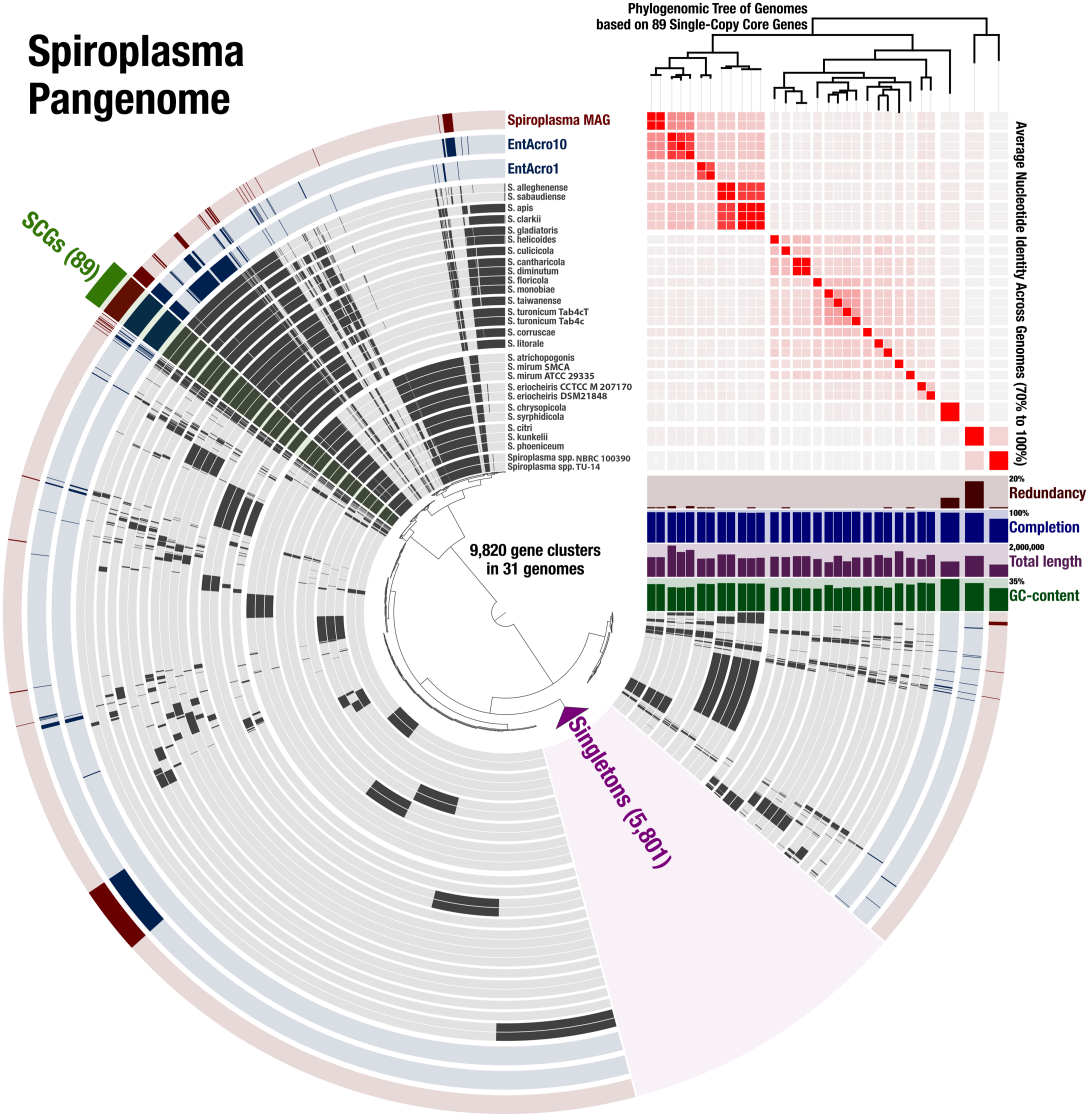
*Spiroplasma.* spp. Have a Large Pangenome. Pangenomic analysis of 28 sequenced *Spiroplasma* spp., and 3 mollicute MAGs (including the WSS-associated *Spiroplasma* MAG) revealing 89 (<1%) single copy core gene clusters, and 5,801 (59%) species-specific gene clusters among 9,820 total gene clusters along with their distribution and average nucleotide identities.

**Figure 3:**
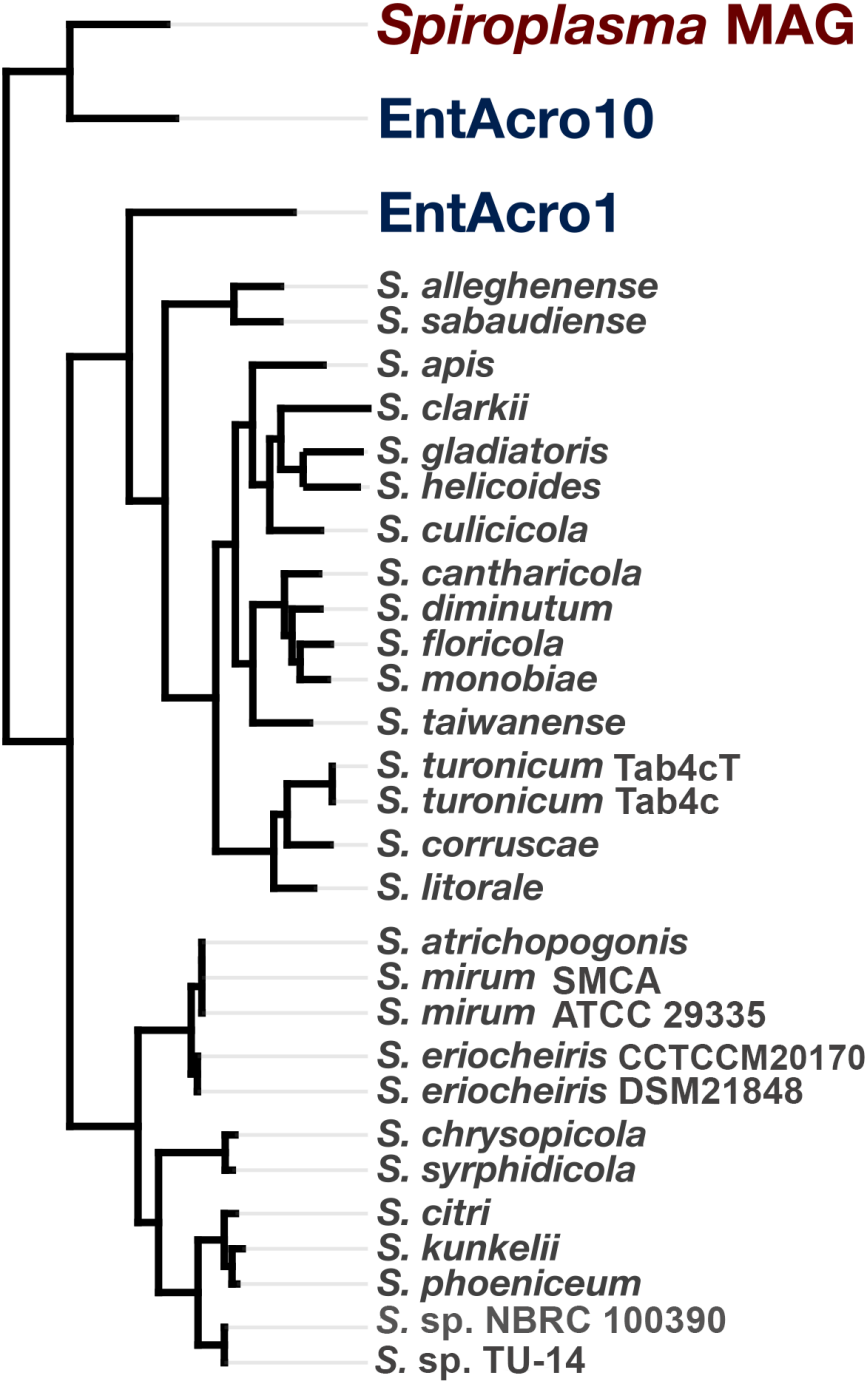
Phylogeny of *Spiroplasma.* sp. WSS shows it belongs to the Ixodetes clade of Mollicutes. A maximum likelihood tree constructed from the 89 single-copy core *Spiroplasma* genes revealed from a pangenomic analysis showing the phylogenomic relationship of 28 sequenced *Spiroplasma* spp., and three mollicute MAGs (including the WSS-associated *Spiroplasma* MAG) and their distribution among the three phylogentic clades: Apis, Citri-Chrysopicola-Mirum (CCM), and Ixodetis.

To determine the relationship of the *Spiroplasma* sp. WSS to previously published *Spiroplasma* genomes, we used a total of 31 genomes that included our MAG, two recently published Entomoplasmatales genomes (Sapountzis et al., 2018), and 28 *Spiroplasma* genomes available from NCBI (Table S1). The *Spiroplasma* pangenome (Fig. 2) revealed a total of 9,820 gene clusters. 89 of these gene clusters were those that represented single-copy core genes of the *Spiroplasma* pangenome (where each genome contributed precisely a single gene), and 5,801 of those were singletons (gene clusters and alignments are reported in Supplemental Information 2). Our phylogenomic analysis using a maximum likelihood model with the 89 single-copy core genes revealed the three distinct clades that corresponded to the known major mollicute clades (Citri-Chrysopicola-Mirum (CCM), Apis, and Ixodetis) (Fig. 3). The distinction of these clades was also supported by differences in average nucleotide identity values between genomes (Fig. 2, Table S2). *Spiroplasma* sp. WSS was most closely related to the recently described Ixodetis member, EntAcro10 (Sapountzis et al., 2018), with both being distinct from all other *Spiroplasma* spp. that were instead distributed in the CCM and Apis clades. Average nucleotide identity (ANI) between *Spiroplasma* sp. WSS and EntAcro10 was just 73% over 34% of the total alignment (Table S2).

## Discussion

This is the first work to describe and characterize the microbiota of the wheat stem sawfly (WSS; *Cephus cinctus*). We show by 16S rRNA gene sequencing, metagenomic data, and SEM imaging that WSS is symbiotically colonized by a novel *Spiroplasma* species of the Ixodetis clade. The *Spiroplasma* species was seen in both adults and larvae that had not yet emerged from wheat stems suggesting the bacterium is obtained early in life either vertically from the female before oviposition or is picked up horizontally from feeding on the inner wheat stem lining. Several less abundant members of the Pasteurellaceae family were also detected, however, due to our whole-WSS processing approach to obtain DNA, it is not clear if these microbes are co-located in the WSS with *Spiroplasma* sp. WSS.

SEM imaging indicated a characteristically long and filamentous but non-helical structure. *Spiroplasma* cells have been shown to vary in morphology based on conditions and age of *in vitro* culture, with older cells or those that have been cultured in sub-optimal conditions tending to lack helical structure (Itoh, Pan & Koshimizu, 1989). Thus, the non-helical structure observed may be an artifact of our inability to cultivate *Spiroplasma* sp. WSS (see supplementary material for further discussion on cultivation efforts). Alternatively, strains of *Spiroplasma* lacking helical structure, regardless of culture conditions have been identified, including a non-helical strain of *S. citri* (Townsend, Burgess & Plaskitt, 1980) and *S. culilicola,* which is also reported to predominantly have a non-helical morphology (Hung et al., 1987). Known morphologies of *Spiroplasma* from the Ixodetis clade, in particular, have been reported to vary widely. For example, *S. platyhelix* exhibits a “kinked” morphology (Townsend, Burgess & Plaskitt, 1980). Nevertheless, further research is necessary to optimize growth conditions for the *Spiroplasma* sp. WSS and subsequently confirm or refute its non-helical morphology.

SEM images also revealed some cells with a Y-shaped morphology. This Y-shape has previously been observed among *S. poulsonii* cells as characteristic of their longitudinal scission method of cell division (Ramond et al., 2016). This observation for *Spiroplasma* sp. WSS therefore adds support to the hypothesis that longitudinal scission is the most prevalent method of cell-division utilized by this bacterial genus (Ramond et al., 2016).

Using advanced metagenomic sequencing, assembly, and binning processes, we were able to obtain genomic insights into *Spiroplasma* sp. WSS. *Spiroplasma* genome sequencing and assembly is notoriously difficult due to an A-T rich nucleotide composition, which for some *Spiroplasma* genomes can be further complicated by repetitive transposon-like elements and plectiviral sequences (Bolaños, Servín-Garcidueñas & Martínez-Romero, 2015; Lo, Gasparich & Kuo, 2015). Additional challenges experienced in completing the *Spiroplasma* sp. WSS genome included its recalcitrance to cultivation and limitations when attempting to enrich *Spiroplasma* DNA from the WSS host and wheat DNA. Nevertheless, the 714 KB draft sequence provides important insight into the WSS symbiont, as well as more broadly into the Ixodetis clade (Bolaños, Servín-Garcidueñas & Martínez-Romero, 2015).

*Spiroplasma* sp. WSS is only the second member of the Ixodetis clade for which genomic data has been obtained, and although phylogenomic data supports the relationship between this genome and the other Ixodetis MAG, EntAcro10, it is clear that there are substantial genetic differences between the two with just 73% ANI between the two genomes. Given the suggested 95% ANI for species boundaries (Konstantinidis, Ramette & Tiedje, 2006), these data suggest that *Spiroplasma* sp. WSS and EntAcro10 represent relatively closely related yet distinct taxa.

Our data indicate *Spiroplasma* sp. WSS has a complete genome size of ∼0.98 Mb, consistent with the small genome size predicted for EntAcro10 (Sapountzis et al., 2018) and the typically small *Spiroplasma* genomes described to date that have ranged from 0.78–2.2 Mb, the upper and lower bounds of which belonging to members of the Ixodetis clade (Bolaños, Servín-Garcidueñas & Martínez-Romero, 2015; Lo, Huang & Kuo, 2016). The draft genome includes evidence for only a single rRNA operon, consistent with all previous *Spiroplasma* genomes, except *S. sabaudiense,* and 23 tRNA genes, approximately the number expected from this level of genome completeness, given other *Spiroplasma* genomes house 29-33 tRNA genes (Ku et al., 2013; Bolaños, Servín-Garcidueñas & Martínez-Romero, 2015; Lo, Gasparich & Kuo, 2015). The *Spiroplasma* genome provides preliminary insight into the nutritional requirements for the bacterium that we hope will help guide future cultivation efforts necessary for taxonomic description and to advance our ability to elaborate the extent of interaction between *Spiroplasma* sp. WSS and the WSS host. Although the genome is only ∼73% complete, its gene content indicates the bacterium can import and phosphorylate sugars via a phosphoenolpyruvate phosphotransferase (PTS) system with pathways for the utilization of G6P, and fructose-6P. Consistently, the utilization of glucose is a universal characteristic of all *Spiroplasma* spp. described to date and fructose utilization is well described in the CCM and Citri clades (Gaurivaud et al., 2000; Whitman, 2011). Genes for the production of lactate and the short chain fatty acids acetate and propanoate suggest these are likely the major fermentative end products. This is also consistent, with lactate and acetate having both previously been observed as the major fermentative end products of *S. citri* (Gaurivaud et al., 2000). Additional carbohydrate substrate utilization pathways may exist within the uncaptured regions of the genome as would be consistent with previous studies that have found horizontal gene transfer events have led to a high diversity of carbohydrate transporters among *Spiroplasma* (Chang et al., 2014; Lo, Gasparich & Kuo, 2015; Lo & Kuo, 2017).

Of potential importance to the symbiosis of *Spiroplasma* sp. WSS with the insect host is the bacterium’s encoded ability to biosynthesize the B vitamins riboflavin (B2), Niacin (B3), and folate (B9). Currently, the available genome sequence for WSS lacks genes necessary for the biosynthesis of most B-vitamins, with only genes for the biosynthesis of pyridoxine (B6) and a partial folate biosynthetic pathway evident ( Text S2 and Fig. S2). The ability of *Spiroplasma* sp. WSS to both biosynthesize and import folate and nicotinamide suggests these vitamins may be of particular importance especially in adult life stages following emergence from the wheat stem, where these B-vitamins are abundant (Kumar et al., 2011). The presence of pathways for the biosynthesis and uptake of folate have been described in several bacterial species, including several pathogens, where the additional ability to uptake folate has been found to confer resistance to antifolate drugs that target the folate biosynthetic and thymidylate pathways (Wang et al., 2004; De Crécy-Lagard et al., 2007; Eudes et al., 2008). Yet the potential benefit of such resistance to either WSS or its *Spiroplasma* symbiont is unclear.

Additionally, the *Spiroplasma* sp. WSS genome was found to encode cardiolipin synthetase and chitinase. Cardiolipin is a component of the *Spiroplasma* membrane and is also found in eukaryotic mitochondria (Paredes et al., 2015). When produced in excess in eukaryotic cells, cardiolipin can promote apoptosis (Gonzalvez & Gottlieb, 2007), and consequently it has been hypothesized that it may play a role in insect defense against nematodes and parasitic wasps due to its toxic nature (Paredes et al., 2015). Chitinase may also defend against parasitic wasps by degrading their chitin-based cuticles, as well as facilitating effective digestion of WSS larva consumed by internecine cannibalism (Buteler et al., 2015). In addition, this ability would also extend to digestion after predation upon vulnerable parasitoids feeding on the paralyzed host, which is an expectation in the population dynamics across trophic levels in this system (Weaver et al., 2005). To date, neither cardiolipin or chitinase have demonstrably been shown to be actively involved in insect defenses against parasitoid wasps or nematodes (for a review of current knowledge on these processes see Ballinger & Perlman (2019). Nevertheless, the putative roles of cardiolipin and chitinase in protecting against parasitic wasps is intriguing because the WSS is commonly parasitized by at least two congeneric species of parasitic wasps: *Bracon cephi* and *B. lissogaster* (Morrill, Kushnak & Gabor, 1998). For each species, WSS larvae are the only known hosts; *B. lissogaster* is more frequently associated with WSS living in native grass stems than *B. cephi,* which primarily parasitizes WSS colonizing wheat (Morrill, Kushnak & Gabor, 1998). The role of this metabolite may be of further importance in consideration of inability of an exotic parasitoid that had not co-evolved with the host to successfully develop on native WSS despite being a strong candidate (Rand et al., 2016; Rand, Waters & Shanower, 2016). We hope these findings will motivate future studies investigating the role of *Spiroplasma* sp. WSS symbiosis in host resistance to *Bracon* spp. wasp parasitism.

The identification of several genes showing homology to the male-killing Spaid gene of *S. poulsonii* may warrant further analysis to determine if *Spiroplasma* sp. WSS may be capable of affecting WSS sex ratios. However, it is noteworthy that all gene-candidates within the *Spiroplasma* sp. WSS genome lacked the deubiquitinase domain that was determined by Harumoto & Lemaitre (2018) to be essential to effective male-killing activity. Further, although a previous study on the sex ratio of WSS larvae determined a female larvae bias in the preferred larger-stemmed wheat, it also found a male-bias of larvae oviposited in smaller-stemmed wheat (Morrill et al., 2000). Nevertheless, beyond Spaid, there appears few examples of ankyrin-repeat containing genes among *Spiroplasma* spp. and so these genes are worthy of further characterization.

Other symbiotic attributes reported among various other *Spiroplasma* spp. described to date, such as improving host defenses against fungal pathogens (Łukasik et al., 2012) cannot be determined because the genetic determinants are yet to be described and phylogeny is not indicative of these traits. For example, this might include defense against a complex of *Fusarium* spp. commonly occurring in wheat that have been shown to kill WSS larvae (Wenda-Piesik et al., 2009). Likewise, the potential for *Spiroplasma* sp. WSS to contribute to the wheat damage associated with WSS, as has been described in corn and citrus (Chen & Liao, 1975; Regassa & Gasparich, 2006; Haselkorn, 2014; Bolaños, Servín-Garcidueñas & Martínez-Romero, 2015) is unknown but deserves attention.

Overall, these findings identify the presence of a previously undescribed *Spiroplasma* symbiont of the WSS, a major wheat pest. Although we have been, thus far, unable to cultivate the bacterium, metagenomic sequencing and binning provided genetic insight into the lifestyle of *Spiroplasma* sp. WSS The genome sequence also provides the first opportunity for comparative genomic analyses of members of the Ixodetis clade of mollicutes and highlights substantial genomic variation among its earliest genome resolved members.

## Conclusion

We set out to identify the symbiotic microbe(s) of WSS and obtain insight into the nature of the insect-microbe relationships so that we could begin to determine if these insect-microbial relationships could be exploited as alternate measures to control WSS damage in crops. Although our ability to determine if the WSS-*Spiroplasma* symbiosis may be manipulated to impact the WSS fitness requires further experimentation, the identification of *Spiroplasma* sp. WSS and greater genetic insight into its metabolism provide a critical first step toward our pursuit of a novel biocontrol approach.

##  Supplemental Information

10.7717/peerj.7548/supp-1 Supplemental Information 1 Supplementary Text Descriptions of Attempts to Isolate and Culture *Spiroplasma* sp. and Metabolic Pathway Analysis Of Draft Wheat Stem Sawfly and *Spiroplasma* sp. Genomes

10.7717/peerj.7548/supp-2 Supplemental Information 2 *Spiroplasma* Pangenome The 9,820 gene clusters determined from pangenomic analyses of 28 Spiroplasma spp. and 3 MAGs and their alignments.

10.7717/peerj.7548/supp-3 Table S1 *Spiroplasma* Genomes Used In Phylogenomic analyses A list of the 28 Spiroplasma species and 3 MAGs downloaded and utilized in our comparative analyses along with their NCBI accession information

10.7717/peerj.7548/supp-4 Table S2 Average Nucleotide Identities of *Sprioplasma* spp Average nucleotide identities from alignments of 89 single copy core genes identified in the pangenome of *Spiroplasma*.

10.7717/peerj.7548/supp-5 Table S3 Culture Metabolomic Analyses Longitudinal metabolomic analyses of cultivation efforts showing metabolites whose concentrations were deferentially altered (highlighted green) and others that were detected.

10.7717/peerj.7548/supp-6 Figure S1 Contig Recruitment By Sample Shows the recruitment of all contigs by sample to each of the three bins, WSS host, wheat, and the *Spiroplasma* sp. symbiont.

10.7717/peerj.7548/supp-7 Figure S2 Metabolic Pathway Overlap of WSS and *Spiroplasma* sp. From Draft Genome Data Metabolic pathways encoded by genes found exclusively in the draft WSS (green) or *Spiroplasma* sp. (red) genome and those in both (orange) are shown on a KEGG map as created by iPATH.

## Additional Information and Declarations

### Field Study Permissions

The following information was supplied relating to field study approvals (i.e., approving body and any reference numbers): Permission to collect the Highland population was obtained from the Lincoln Ranger District. There are no formal permits required for this because it is an incidental population of a highly abundant agricultural pest. The lowland populations were collected on land cultivated by Wheat Montana Farms with permissions obtained from Dean Folkvord and on land cultivated by Matt Flikkema.

### DNA Deposition

The following information was supplied regarding the deposition of DNA sequences: 16S rRNA sequences are available in the Sequence Read Archive: SRP108219. Metagenomic sequences are available in the Sequence Read Archive: SRP108220. Additional sequence data generated by Illumina HiSeq as part of the WSS genome project (Robertson et al., 2018) are available at: SRS694145. The metagenomic assembled genome is available in GenBank under bioproject PRJNA540284 and is available publicly at Patric with the genome ID 2132.146 (Spiroplasma WSS).
