# Supplementary material for: Genome-resolved insights into a novel Spiroplasma symbiont of the Wheat Stem Sawfly (Cephus cinctus)
Source: PeerJ. 2019 Aug 27;7:e7548. doi: 10.7717/peerj.7548 (PMC6716498; doi:10.7717/peerj.7548)
Supplement: Supplemental Information 1 — Descriptions of Attempts to Isolate and Culture Spiroplasma sp. and Metabolic Pathway Analysis Of Draft Wheat Stem Sawfly and Spiroplasma sp. Genomes [file peerj-07-7548-s001.pdf]

Supplementary Text 1: Attempts to Isolate and Culture *Spiroplasma* sp.

Spiroplasma are known to be fastidious to culture. In efforts to isolate the sawfly associated *Spiroplasma*, one hundred larval specimens were washed in 70% ethanol followed by rinsing with sterile water. Based on the protocol used by (Tully, 2012), larvae were then lightly ground with a mortar and pestle in 5 ml sterile PBS containing 7.5 percent bovine plasma albumin (pH 7.5) under a flow hood. The entire solution was filtrated through a disposable 450 nm nitrocellulose filter in order enrich the bacteria in the cell lysate for *Spiroplasma* and remove other bacterial species (Nai et al., 2014).

Immediately following filtration, 100 µl of the cell lysate filtrate was inoculated into 5 ml each of three different media types (prepared as per ATCC instructions) previously used to cultivate *Spiroplasma* species: M1D media (ATCC 798) (Hackett et al., 1996), Singh's media (ATCC 1541) (Williamson et al., 1997), and SP-4 (ATCC 988) (Abalain-Colloc, and1988, 1988). The media were then incubated at 30°C and monitored daily for a change in pH as indicated visually by a change in media color from red to yellow (via phenol red) as an indication of microbial metabolism.

Spiroplasma cultures indicated brief periods of growth to extremely low densities of biomass in both Singh's and SP-4 media. Sparsely distributed, small non-Brownian motion 'wriggling' bacteria (<5 nm) were observed by microscope using 100x magnification during the first 2-3 days of each culturing attempt, but were not usually observed after four days. This may indicate that both media types were sufficient to prolong viability of the bacterium, but that neither media types were sufficient for growth of the isolated bacteria. Attempts were made to improve cultivation through the incorporation of additional nutrients. However, only the addition of cholesterol (10µg/ml) to Singh's media resulted in any observable improvement, and extended the culturing time to ~4-5 days, albeit with no visible increase in cell density.

In a final effort to identify potential nutrient deficits in the culturing media, samples of the three replicate cultures in Singh's media supplemented with cholesterol (10µg/ml) were collected at 0, 1, 2, and 4 days post-inoculation for metabolomic analysis. Samples were dried and derivatized with 50 µL methoxyamine hydrochloride (Sigma-Aldrich, MO, USA) (40 mg mL-1 in pyridine) for 60 min at 400°C, then with 50 µL MSTFA+1%TMCS (Thermo, MA, USA) at 500°C for 120 min, and following 2-hour incubation at room temperature. Then, 5 µL of the internal standard (hentriacontanoic acid, 10 mg mL-1) was added to each sample prior to derivatization. Metabolite profiles were acquired using a GC-MS system (Agilent Inc, CA, USA) consisting of an Agilent 7890 gas chromatograph, an Agilent 5975 MSD and a HP 7683B autosampler. Gas chromatography was performed on a ZB-5MS (60m×0.32mm I.D. and 0.25 µm film thickness) capillary column (Phenomenex, CA, USA). The inlet and MS interface temperatures were 2500 °C, and the ion source temperature was adjusted to 2300°C. An aliquot of 1 µL was injected with the split ratio of 15:1. The helium carrier gas was kept at a constant flow rate of 2 ml min-1. The temperature program was: 5-min isothermal heating at 700°C, followed by an oven temperature increase of 50C min-1 to 3100°C and a final 10 min at 3100°C. The mass spectrometer was operated in positive electron impact mode (EI) at 69.9 eV ionization energy at m/z 30-800 scan range. The spectra of all chromatogram peaks were evaluated using the AMDIS 2.71 (NIST, MD, USA) using a custom-built database (460 unique metabolites). All known artificial peaks were identified and removed prior to data mining. To allow comparison between samples, all data were normalized to the internal standard in each chromatogram and the sample volume. The instrument variability was within the standard acceptance limit (5%).

Eighteen metabolites were observed to either consistently increase or decrease in the cultures (Table S3). These included decreases in pyruvate, trehalose, serine, and cysteine over time and increases in the amino acids alanine, arginine, aspartic acid, proline, serine, tyrosine, valine,

and pyroglutamic acid, as well as uridine, xanthine, allantoin, glycolic acid, 2-aminoethylglycerophosphate, 3,4-dihydroxybutanoic acid, and galactose.

These observations, even when using large cultivation volumes (2 L) under the most optimal conditions, did not enable us to obtain sufficient DNA for full length 16S rRNA gene sequencing to confirm the cultivate was the *Spiroplasma* sp. or otherwise identify the contaminant. Therefore, it is unclear if these metabolomic results are driven by the *Spiroplasma* sp., a contaminant, and/or spontaneous changes in the metabolome over the period of incubation. Retrospective analyses following genome generation show these metabolite changes do not perfectly align with available genomic data or with characteristic *Spiroplasma* traits, for example *Spiroplasma* spp. are metabolically defined by their ability to ferment glucose a feature that was also evident in the genome[30], yet no decrease in glucose was observed. They are also typically able to hydrolyze arginine but unable to hydrolyze urea[30]. Therefore, these results are only provided in supplementary in the hope they may aid additional efforts to cultivate this bacterium.

Supplementary Text 2: Metabolic Pathway Analysis Of Draft Wheat Stem Sawfly and *Spiroplasma* sp. Genomes

To obtain preliminary insight into potential functional interactions, both the WSS host and *Spiroplasma* sequence bins were annotated to derive Kegg Ontologies and these were mapped to metabolic pathways (Fig. S2). Based on the available genome data for WSS from both the raw sequence files from the WSS genome sequencing effort available in Genbank under accession #SRS694145 and our own sequencing contributions following binning.

Among existing sequence data, the *Spiroplasma* sp. endosymbiont encodes amino acid, vitamin, glutathione, and glycerolipid and glycerophospholipid metabolic capabilities not observed in the draft WSS genome (Fig. S2).

Comparative analysis with the host WSS genome indicated multiple potential metabolic interactions between the host and endosymbiont, including for glucose, amino acid, vitamin, glutathione, and glycerolipid and glycerophospholipid metabolic capabilities.

Literature Cited

Abalain-Colloc ML, and LROS, 1988 1988. Spiroplasma taiwanense sp. nov. from *Culex tritaeniorhynchus* mosquitoes collected in Taiwan. *International Journal of Systematic Bacteriology* 38:103–107.

Hackett KJ, Whitcomb RF, French FE, Tully JG, Gasparich GE, Rose DL, Carle P, Bové JM, Henegar RB, Clark TB, Konai M, Clark EA, Williamson DL 1996. *Spiroplasma corruscae* sp. nov., from a firefly beetle (Coleoptera: Lampyridae) and tabanid flies (Diptera: Tabanidae). *International Journal of Systematic Bacteriology* 46:947–950. DOI: 10.1099/00207713-46-4-947.

Nai Y-S, Su P-Y, Hsu Y-H, Chiang C-H, Kim JS, Chen Y-W, Wang C-H 2014. A new spiroplasma isolate from the field cricket (*Gryllus bimaculatus*) in Taiwan. *Journal of Invertebrate Pathology* 120:4–8. DOI: 10.1016/j.jip.2014.04.006.

Tully J 2012. *Methods in Mycoplasmology V2*: Diagnostic Mycoplasmology Elsevier.

Williamson DL, Adams JR, Whitcomb RF, Tully JG, Carle P, Konai M, Bové JM, Henegar RB 1997. *Spiroplasma platyhelix* sp. nov., a new mollicute with unusual morphology and genome size from the dragonfly *Pachydiplax longipennis*. *International Journal of Systematic Bacteriology* 47:763–766. DOI: 10.1099/00207713-47-3-763.
