# Supplementary figures and images for: Genome-resolved insights into a novel Spiroplasma symbiont of the Wheat Stem Sawfly (Cephus cinctus)
Source: PeerJ. 2019 Aug 27;7:e7548. doi: 10.7717/peerj.7548 (PMC6716498; doi:10.7717/peerj.7548)

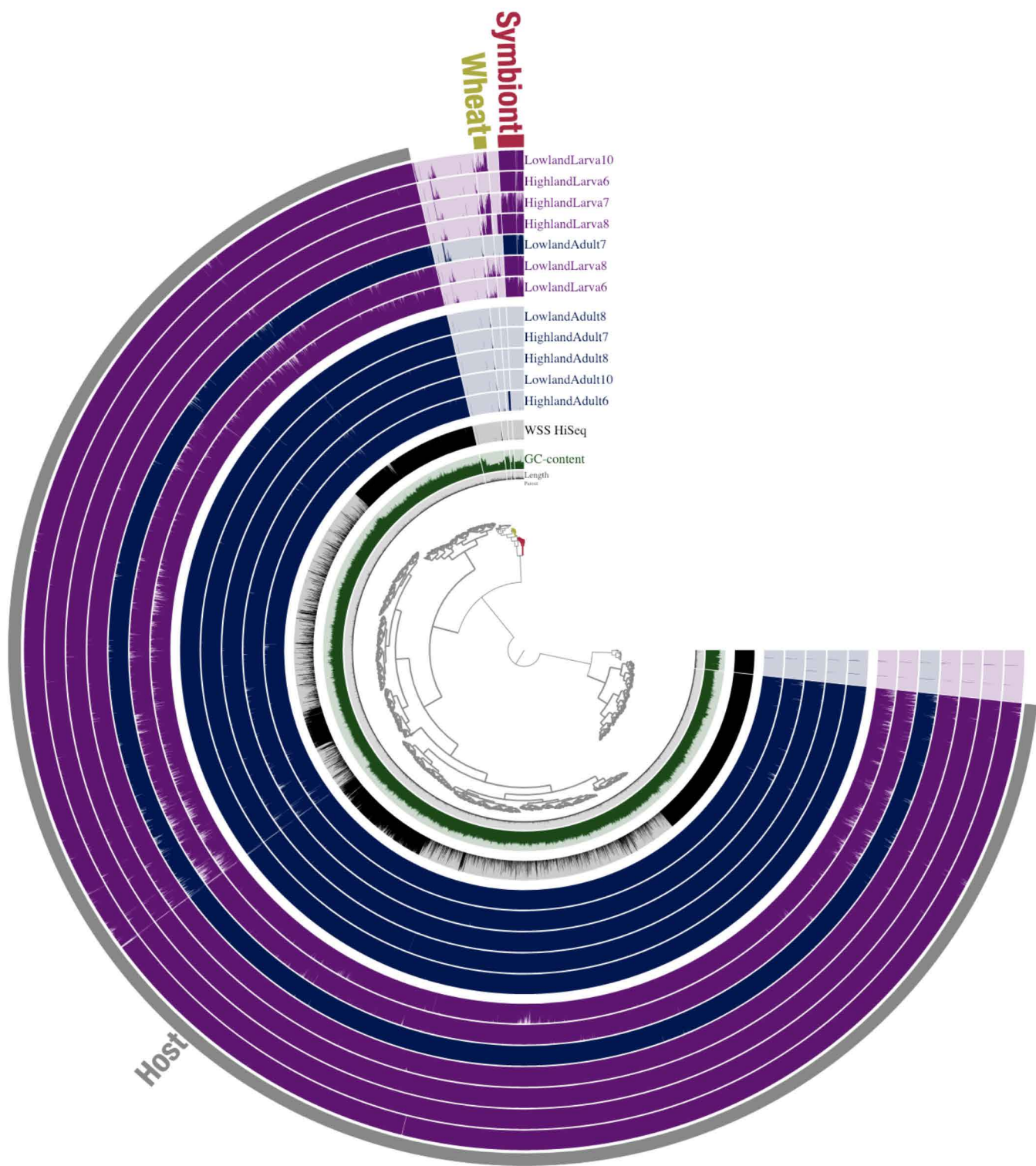

Supplement: Figure S1 — Shows the recruitment of all contigs by sample to each of the three bins, WSS host, wheat, and the Spiroplasma sp. symbiont. [file peerj-07-7548-s006.pdf]

# Glycerolipid & Glycerophospholipid metabolism

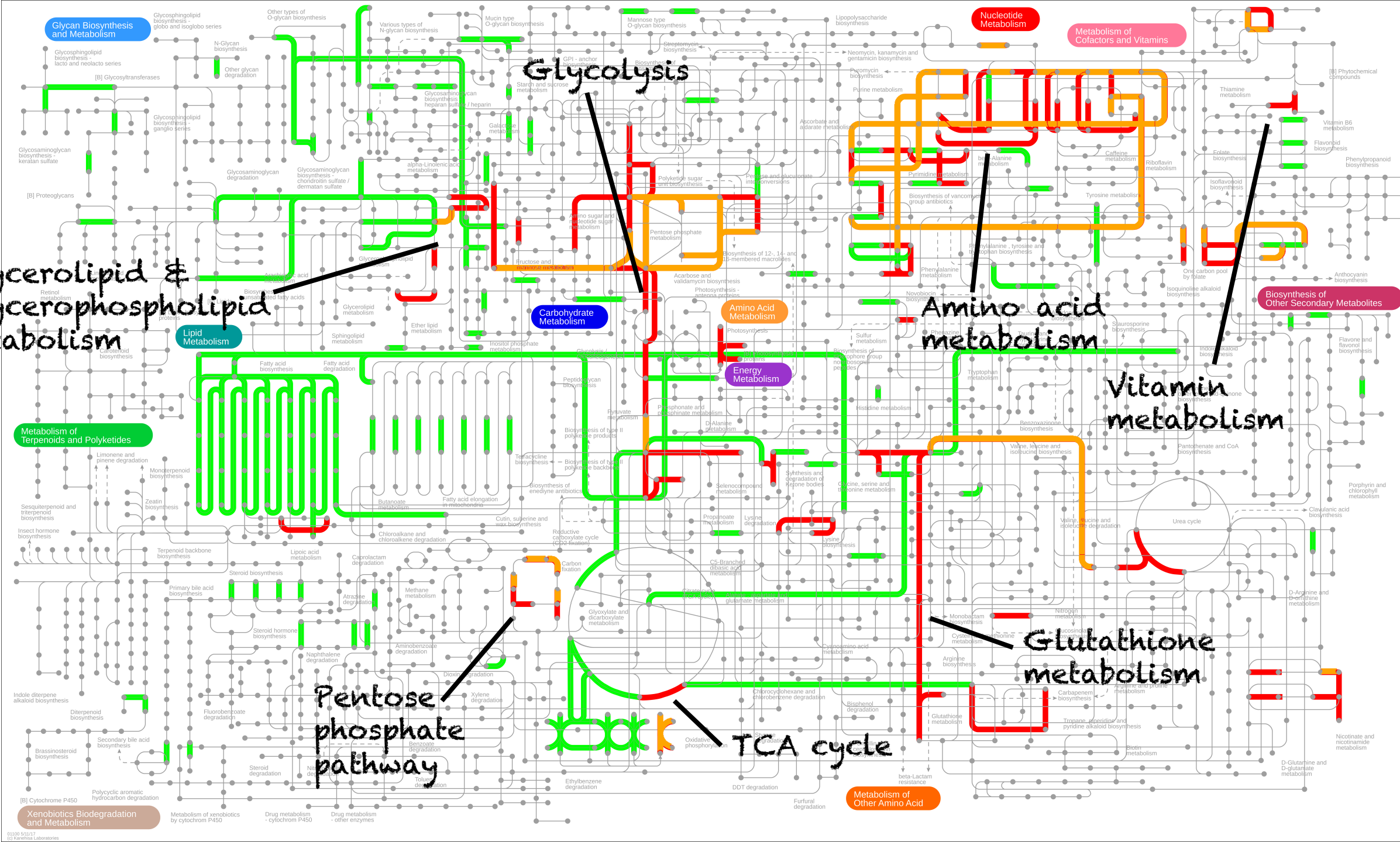

Supplement: Figure S2 — Metabolic pathways encoded by genes found exclusively in the draft WSS (green) or Spiroplasma sp. (red) genome and those in both (orange) are shown on a KEGG map as created by iPATH. [file peerj-07-7548-s007.pdf]
